# Factors affecting the determination of cerebrovascular reactivity

**DOI:** 10.1002/brb3.275

**Published:** 2014-08-26

**Authors:** Rosemary E Regan, Joseph A Fisher, James Duffin

**Affiliations:** 1 Department of Physiology, University of TorontoToronto, ON, M5S 1A8, Canada; 2 Department of Anaesthesiology, University of TorontoToronto, ON, Canada; 3 University Health NetworkToronto, ON, Canada

**Keywords:** Blood pressure, cerebral blood flow, CO_2_, humans, hypoxia

## Abstract

**Background and Purpose:**

Cerebrovascular reactivity (CVR), measures the ability of the cerebrovasculature to respond to vasoactive stimuli such as CO_2_. CVR is often expressed as the ratio of cerebral blood flow change to CO_2_ change. We examine several factors affecting this measurement: blood pressure, stimulus pattern, response analysis and subject position.

**Methods:**

Step and ramp increases in CO_2_ were implemented in nine subjects, seated and supine. Middle cerebral artery blood flow velocity (MCAv), and mean arterial pressure (MAP) were determined breath-by-breath. Cerebrovascular conductance (MCAc) was estimated as MCAv/MAP. CVR was calculated from both the relative and absolute measures of MCAc and MCAv responses.

**Results:**

MAP increased with CO_2_ in some subjects so that relative CVR calculated from conductance responses were less than those calculated from CVR calculated from velocity responses. CVR measured from step responses were affected by the response dynamics, and were less than those calculated from CVR measured from ramp responses. Subject position did not affect CVR.

**Conclusions:**

(1) MAP increases with CO_2_ and acts as a confounding factor for CVR measurement; (2) CVR depends on the stimulus pattern used; (3) CVR did not differ from the sitting versus supine in these experiments; (4) CVR calculated from absolute changes of MCAv was less than that calculated from relative changes.

## Introduction

Cerebrovascular reactivity (CVR) is measured as the ratio of the change in cerebral blood flow (CBF) in response to a change in a vasoactive stimulus (Willie et al. 2014). The CBF response, or its surrogate, can be estimated using transcranial Doppler (TCD) measures of flow velocity in major blood vessels (Willie et al. 2011). Alternatively, detailed maps of CBF responses can be obtained by estimating CBF using Blood Oxygen Level Dependent (BOLD) functional magnetic resonance imaging (fMRI). As discussed elsewhere, CO_2_ is an ideal choice for the vasoactive stimulus (Fierstra et al. 2013). Such measurements not only inform the physiological regulation of cerebral blood flow (e.g. Willie et al. 2012), but can also be used to detect pathophysiology (e.g. Mandell et al. 2008b), and to monitor the efficacy of surgical interventions (e.g. Han et al. 2011). In this paper, we examine several factors that influence the determination of CVR.

Cerebral blood flow is affected by perfusion pressure (Panerai et al. 1999; Dineen et al. 2010; Lucas et al. 2010), metabolism (Iadecola and Nedergaard 2007; Attwell et al. 2011; Paulson et al. 2011) and CO_2_ (Ainslie and Burgess 2008; Battisti-Charbonney et al. 2011). The response to CO_2_ is thought to be mediated by a direct action of CO_2_ on cerebral arteriolar vessels via a change in vessel wall pH to decrease cerebral flow resistance (Lassen 1968; Kontos et al. 1977; Tian et al. 1995). Similarly, pressure autoregulation also acts to control CBF by altering cerebral flow resistance, so that CO_2_ and pressure autoregulation compete for the control of vessel caliber (Harper and Glass 1965).

Increasing CO_2_ may also increase systemic blood pressure and in doing so increase cerebral perfusion pressure. In a recent study of subjects seated at rest Battisti-Charbonney et al. (2011) observed changes in mean arterial pressure (MAP) with increasing CO_2_. They found that below a threshold CO_2_ tension, MAP changed little with CO_2_ but above the threshold MAP increased linearly with CO_2_ tension. In the range of constant MAP, CBF exhibited a sigmoidal variation with CO_2_, approaching a plateau at the higher CO_2_ levels. However, on further increases in CO_2_, the relationship changed and MAP and CBF increased in tandem. Thus, when CO_2_ vasodilation has reached its limit, increases in perfusion pressure increase CBF passively. As we showed in these experiments, this interaction of MAP and CO_2_ in controlling CBF makes it difficult to distinguish between the effects of CO_2_ on vasoreactivity and those mediated by changes in perfusion pressure, either via autoregulation vasoconstriction or passive perfusion.

Cerebrovascular reactivity measurements using Doppler ultrasound may be made either sitting (Ainslie and Burgess 2008) or supine (Claassen et al. 2007), but when CBF is estimated using BOLD fMRI, the supine position is required. Since brain perfusion pressure may differ between these two positions due to gravity and changes in MAP (T-M et al. 2008), it is important to determine if CVR also differs with position. A single study using a carbogen stimulus (McDonnell et al. 2013) reported a difference in CVR measurement reliability due to body position. We therefore compared CVR measured while sitting with CVR measured when supine in these experiments.

As has been demonstrated, the stimulus pattern affects the CVR measurement (Sobczyk et al. 2014). Due to the sigmoidal nature of the vasodilatory response, the CVR measured will be affected by the range of the hypercapnic stimulus; CVR decreases with higher stimulus levels as the vasodilatory limit is reached. As we demonstrate in these experiments, CVR is also affected by the dynamic aspects of the CO_2_ stimulus and the CBF response. For example, the time course of CBF response to a step increase in CO_2_ varies between subjects (Regan et al. 2013) so that in some subjects the full response takes time to be reached. By contrast, a gradual ramping up of Pco_2_ provides sufficient time for the CBF response to attain a steady state with respect to the Pco_2_. The effects of different stimulus patterns on CVR measurements resulting from rebreathing, breath-holding, hyperventilation, and carbogen inhalation (Totaro et al. 1999) have been studied previously. However, these stimuli all have similar slow changes and do not include fast changes in CO_2_ where the dynamics of the response are important (Poulin et al. 1996) in determining CVR. Indeed, we know of no studies that have considered the effects of stimulus dynamics on the CVR measured. We therefore compared CVR calculated from step versus ramp stimulus patterns, as well as comparing CVR between fast and slow step responses.

Finally, the response analysis methods that were used affect the resulting CVR. For time-domain analyses, applying a linear or nonlinear (e.g. sigmoid) fit to the CBF responses alters the resulting CVR. We note that for these analyses, the CBF responses may be measured in absolute or relative values. We also introduce the possibility of using frequency domain analysis to calculate CVR with transfer function analysis (TFA), such as that used in analyzing dynamic pressure autoregulation (Tzeng et al. 2012). TFA provides the estimates of both the magnitude of the response to CO_2_ as well as the phase relationship to the stimulus, which would reflect the speed of the response. Measuring not only the magnitude of the response but also an indication of its speed may be of clinical benefit (Conklin et al. 2010; Regan et al. 2013).

Our aim in these experiments was, therefore, to investigate how changes in MAP, and different body positions, stimulus patterns and analysis techniques affect the calculation of CVR. We used CO_2_ as the stimulus and TCD measurement of the middle cerebral artery velocity (MCAv) as the response.

## Methods

### Subjects and ethical approval

These studies conformed to the standards set by the latest revision of the Declaration of Helsinki. Nine (4 mol/L) healthy normotensive, nonsmoking subjects of mean (SD) age 27 (4) years participated in this study after approval from the Research Ethics Board at the Toronto General Hospital (University Health Network) and written informed consent from each of the subjects. We note that our subjects were considered healthy but were not subjected to examination of the neck vessels to exclude the presence of carotid steno-occlusive disease. They were not taking any medication other than oral contraceptives, and had no history or symptoms of cardiovascular, cerebrovascular, or respiratory diseases. In addition, they abstained from caffeinated or alcoholic beverages and vigorous exercise for at least 12 h before the study.

### Apparatus

Subjects were fitted with a face mask, connected to the breathing circuit via a mass flow sensor (AWM720P1 Airflow, Honeywell; Freeport, IL) to monitor ventilation. Beat-by-beat middle cerebral artery flow velocity was measured using bilateral transcranial Doppler (ST3 Transcranial Doppler, Spencer Technologies; Seattle, WA) at 2 MHz and sampled at 125 Hz. Beat-by-beat MAP and HR were determined by finger plethysmography (Nexfin, BMYE; Amsterdam, The Netherlands) sampled at 200 Hz. Tidal gas was sampled and analyzed for the partial pressures of CO_2_ and O_2_ (RespirAct™, Thornhill Research Inc., Toronto, Canada), and recorded at 20 Hz. Each of these instruments saved a digital record for later analysis.

### Protocol

**Figure 1:**
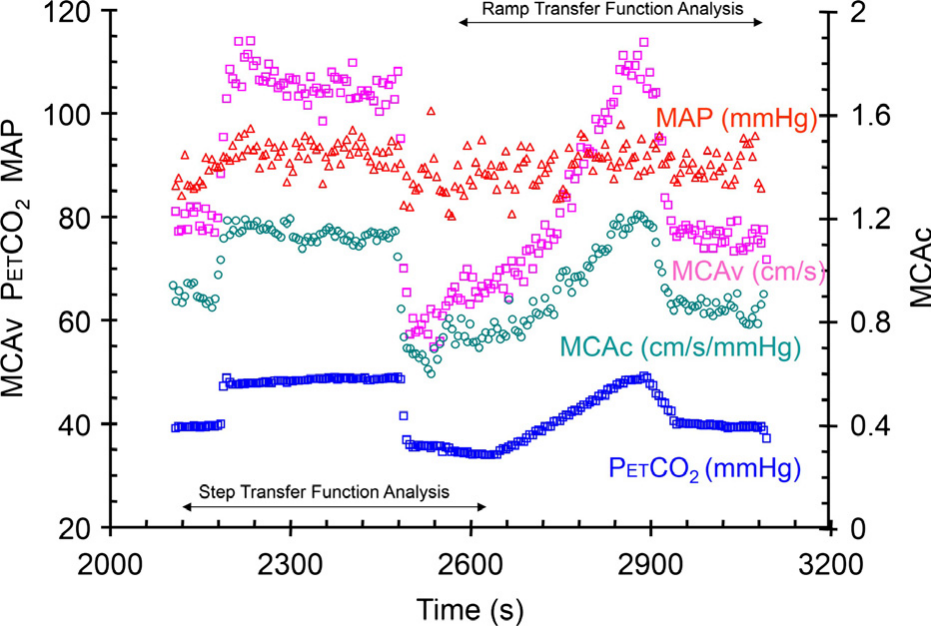
Test results from subject 6 seated, showing breath-by-breath measures of PetCO_2_, MCAv, MCAc and MAP. The axes for each variable are as indicated, and the units are noted with the variable labels. The data selected for transfer function analysis are indicated.

We measured the responses of MCAv, MAP and HR to the following sequence of changes in end-tidal CO_2_ tension (PetCO_2_) while maintaining O_2_ tensions at resting levels (Fig. 1): 2 min at a baseline PetCO_2_ of 40 mmHg, a step increase to 10 mmHg above baseline PetCO_2_ for 5 min, a step decrease to baseline PetCO_2_ and a 2 min ramp decrease from baseline PetCO_2_ to 5 mmHg below baseline, a 4 min ramp increase in PetCO_2_ from 5 mmHg below baseline to 10 mmHg above baseline, followed by a step decrease to baseline PetCO_2_ for 2 min. Control of PetCO_2_ was achieved by prospective targeting, using a sequential gas delivery (SGD) circuit and a computer-driven gas blender (RespirAct™; Thornhill Research Inc.); a method described by (Slessarev et al. 2007). This methodology has been shown to equilibrate arterial Pco_2_ (PaCO_2_) and PetCO_2_ so that the stimulus patterns applied are those of PaCO_2_ (Ito et al. 2008).

Subjects were asked to breathe in time to a metronome at a frequency of 15 b/min and empty the inspiratory bag of the SGD circuit on each breath, thereby preventing rapid changes in ventilation. All nine subjects were tested, once while seated in a comfortable chair and once while lying supine on a bed. The order of the two tests was randomized, and at least 20 min of rest occurred between the two tests. The positions were assumed at least 5 min before measurements began.

### Data analysis

For each test, beat-by-beat values of MAP and HR and 4-sec averages of MCAv were time aligned with breath-by-breath PetCO_2_ and PetO_2_ measures, and breath-by-breath values for MAP, HR and MCAv were calculated as averages of the within-breath measures. Conductance MCAc was calculated as MCAv/MAP for each breath. Baseline measures were determined as the average of the 2-min initial period of the protocol. Relative breath-by-breath MCAv and MCAc measures during the tests were then expressed as percent differences from baseline. Both the breath-by-breath relative changes and the absolute changes in MCAv and MCAc with PetCO_2_ were examined to calculate CVR for the step and ramp stimuli.

CVR was calculated from the step responses in two ways to take into account their dynamic aspects; (All; every hypercapnic breath) the slope of the linear regression of all breath-by-breath values of MCAv and MCAc from the start of baseline to the end of the 5-min step increase in PetCO_2_, and (SS; Steady state) the slope of the linear regression of breath-by-breath values including all baseline breaths, but only the breaths from the final 2 min of the 5-min step increase in PetCO_2_ (where the final value was reached). In addition, the changes in blood pressure during the step changes of PetCO_2_ were measured and classified according to whether or not a threshold increase of 10 mmHg was exceeded. Finally, the speed of the MCAv response was determined by fitting an exponential rise, and classified as slow when the time constant exceeded 5 sec and fast when less.

The ramp responses included all breath-by-breath values of MCAv and MCAc between the minimum and maximum PetCO_2_ of the ramp stimulus. CVR was calculated in two ways; (Lin) the slope of the linear regression, and (Sig) the slope of the sigmoid function, a + (b/(1 + exp(−(x−c)/d))), fitted to the data (Levenburg–Marquard algorithm) at the baseline PetCO_2_. In addition, the changes in blood pressure during the ramp changes of PetCO_2_ were examined and fitted with two linear segments above and below a threshold determined as the best fit.

Finally, we introduced the use of transfer function analysis (TFA) to calculate a TFA CVR from the gain function. Breath-by-breath step and ramp responses were selected from the recordings to obtain 500 sec samples (as indicated in Fig. 1). These were then resampled at 0.5 Hz. Transfer function analysis was based on the Welch algorithm (5 segments with 50% overlap); using Fast Fourier transforms of each nonwindowed segment were averaged to calculate gain, phase and coherence. TFA CVR was estimated from the gain function averaged between 0 and 0.03 Hz.

All of these analyses were assisted by specially written software (LabVIEW, National Instruments, Texas). CVR values calculated from both MCAv and MCAc responses and were compared (SigmaPlot 12.5, Systat Software, San Jose, CA) between various measures with repeated measure analyses of variance (rmANOVA). Where factors were found to be significant, post hoc all pairwise multiple comparisons were made using the Holm–Sidak method. The correlations between step (SS) and ramp CVR (Lin) and TFA CVR values were examined with the Pearson product moment.

## Results

### General considerations

All subjects completed all tests except for subject 7 where the supine ramp response was lost due to a technical failure. Figure 1 shows example recordings from a typical test. In this subject, MCAv and MCAc closely follow the changes in PetCO_2_, and MAP varies only slightly with PetCO_2_.

The step stimuli baseline and hypercapnic portions showed little variation, with the overall mean breath-by-breath variability, expressed as standard deviation, less that 1.6 mmHg for PetCO_2_ and 5.2 mmHg or less for PetO_2_. The differences in PetCO_2_ and PetO_2_ between adjacent breaths, expressed as the overall mean standard deviation, were also small; 1.0 mmHg or less and 2.2 mmHg or less, respectively. That there was little drift during the hypercapnic period of the step stimulus was indicated by the mean of the between breath differences, which was close to zero for both PetCO_2_ and PetO_2_. We concluded that stimulus drift and variation during the step tests was minimal and could not account for the response patterns observed.

Baseline values of MAP, MCAv, and MCAc (Table 1) were tested for differences between sitting and supine positions, and although the mean values for MAP were increased and those for MCAv decreased in the seated position, they were not different. Only baseline MCAc values were different (*P* = 0.007; 1-way rmANOVA); higher in the supine position.

**Table 1:** Mean (SD) baseline values. Significantly different measures are bold and italicized

Position	MAP, mmHg	MCAv, cm/s	MCAc, cm/s/mmHg
Supine	87.3 (20.2)	80.4 (20.6)	***0.93 (0.19)***
Sitting	96.0 (14.8)	77.1 (20.3)	***0.80 (0.17)***

### CVR determined from step increases in CO_2_

Table 2 details the characteristics of the step responses in terms of the changes in MAP and the speed of the response. The latter was measured as the time constant of an exponential rise fitted to the MCAv response. Table 3 shows the CVR values calculated from the MCAv and MCAc responses.

**Table 2:** Step response characteristics. The changes in blood pressure (MAP) and the time constant of the exponential rise fitted to the MCAv response

Subject	Position	Time Constant sec	Time Constant slow ≥ 5 sec	MAP Baseline mmHg	MAP Step mmHg	MAP Increase mmHg	MAP Increase > 10 mmHg
S1	Supine	6.23	Slow	132.7	139.6	6.86	No
S1	Sitting	3.22	Fast	107.8	122.4	14.64	Yes
S2	Supine	1.93	Fast	80.4	86.5	6.10	No
S2	Sitting	3.93	Fast	88.5	95.4	6.89	No
S3	Supine	9.07	Slow	91.5	100.9	9.39	No
S3	Sitting	42.23	Oscillation	104.8	106.1	1.32	No
S4	Supine	6.01	Slow	80.7	109.1	28.41	Yes
S4	Sitting	5.38	Slow	116.2	137.6	21.40	Yes
S5	Supine	5.54	Fast	74.2	80.8	6.58	No
S5	Sitting	2.91	Fast	84.2	90.7	6.46	No
S6	Supine	3.05	Fast	81.4	84.8	3.39	No
S6	Sitting	2.17	Fast	88.1	92.5	4.44	No
S7	Supine	2.72	Fast	103.9	121.7	17.79	Yes
S7	Sitting	2.63	Fast	113.7	124.8	11.13	Yes
S8	Supine	2.69	Fast	66.4	66.5	0.08	No
S8	Sitting	14.16	Oscillation	86.6	92.1	5.57	No
S9	Supine	2.87	Fast	74.2	75.0	0.84	No
S9	Sitting	4.00	Fast	74.6	76.1	1.52	No

**Table 3:** Step response measures of relative (Rel, %/mmHg) and absolute (Abs, cm/s/mmHg) CVR values using the entire hypercapnic period (All) or the steady state portion of the hypercapnic period (SS)

		MCAv				MCAc			
			
		All		SS		All		SS	
					
Subject	Position	Rel	Abs	Rel	Abs	Rel	Abs	Rel	Abs
S1	Supine	4.11	4.25	5.33	5.51	3.16	0.025	4.13	0.032
S1	Sitting	4.19	3.91	4.47	4.16	2.41	0.021	2.70	0.023
S2	Supine	3.07	1.64	3.01	1.61	1.60	0.011	1.66	0.011
S2	Sitting	2.82	1.61	2.99	1.71	1.99	0.013	2.12	0.014
S3	Supine	6.10	4.34	6.43	4.58	4.63	0.036	4.52	0.035
S3	Sitting	3.01	2.18	3.52	2.55	2.91	0.020	3.20	0.022
S4	Supine	2.58	1.90	2.68	1.97	−0.58	−0.005	−0.80	−0.007
S4	Sitting	2.56	1.97	2.53	1.95	0.72	0.005	0.54	0.004
S5	Supine	3.26	2.71	3.43	2.85	2.14	0.024	2.08	0.023
S5	Sitting	3.12	2.60	3.01	2.51	2.18	0.022	2.10	0.021
S6	Supine	3.54	3.05	3.46	2.98	2.94	0.031	2.68	0.028
S6	Sitting	3.55	2.84	3.39	2.71	2.86	0.026	2.70	0.025
S7	Supine	2.14	2.54	2.07	2.46	0.46	0.005	0.46	0.005
S7	Sitting	2.48	2.72	2.54	2.78	1.60	0.015	1.73	0.017
S8	Supine	3.80	2.92	3.63	2.78	3.97	0.046	3.89	0.045
S8	Sitting	2.87	2.38	3.15	2.62	2.05	0.020	2.19	0.021
S9	Supine	3.33	1.93	3.33	1.93	3.13	0.025	3.02	0.024
S9	Sitting	2.41	0.92	2.52	0.97	2.17	0.011	2.22	0.011

### The effect of MAP increases on the measurement of relative CVR

We examined the relative CVR values to discover whether or not they differed when MAP increased using a 2-way ANOVA with factors response (MCAv vs. MCAc) and MAP increase (yes vs. no), treating all tests as independent. Table 4 shows the results of this analysis. CVR calculated from MCAc responses were less than those calculated from MCAv responses for both All and SS determinations when MAP increased. While MAP increase was not a significant factor for CVR calculated from the MCAv responses (*P* = 0.173 and 0.164 for All and SS, respectively), it was for CVR calculated from MCAc responses (*P* ≤ 0.001 and 0.001 for All and SS, respectively). CVR values calculated from MCAc were considerably lower when MAP increased (mean [SD] = 0.92 [1.14] vs. 2.75 [0.86] %/mmHg for the All determination and 0.93 [1.34] vs. 2.81 [0.9] %/mmHg for the SS determinations).

**Table 4:** Mean (SD) CVR calculated from the MCAv and MCAc steady state (SS) relative (Rel, %/mmHg) responses where MAP increased > 10 mmHg during the test or not. The corresponding *P* values are the result of 2-way rmANOVA testing with all pairwise multiple comparisons using the Holm–Sidak method. Significantly different measures are bold and italicized

MAP	All			SS		
		
Increase	MCAv	MCAc	*P*	MCAv	MCAc	*P*
Yes	2.79 (0.80)	0.92 (1.14)	***0.003***	2.86 (0.93)	0.93 (1.34)	***0.005***
No	3.46 (0.91)	2.75 (0.86)	0.054	3.63 (1.06)	2.81 (0.90)	***0.05***
*P*	0.173	***<0.001***		0.164	***0.001***	

**Figure 2:**
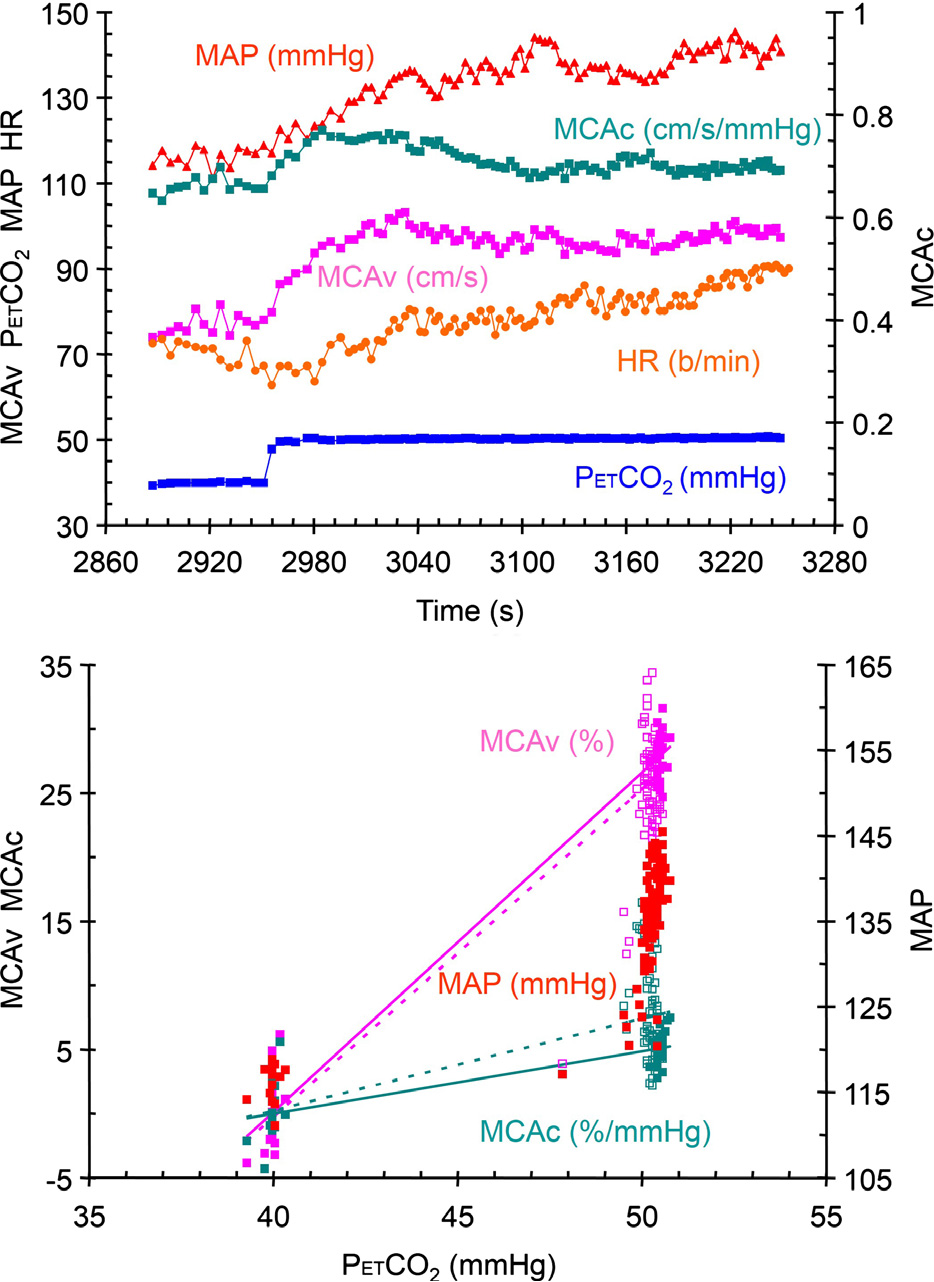
Step test results from subject 4 seated. The top graph shows the breath-by-breath measures of PetCO_2_, MCAv, MCAc, MAP and HR during the step change in PetCO_2_. The lower graph shows the relative MCAv and MCAc responses, and the MAP response to PetCO_2_. The lines in these graphs show the various analyses used to calculate CVR (see text). The SS analysis is indicated by the solid line and filled symbols, and the All analysis is indicated by the dotted line and open symbols. The axes for each variable are as indicated and the units are noted with the color-coded variable labels.

With such a large decrease in CVR conductance (MCAc response) when MAP increased (as illustrated in Fig. 2), we concluded that the CVR values measured during an increase in MAP were confounded such that they did not measure the vasoreactivity to CO_2_. We elaborate on this reasoning in the discussion. Consequently, further examinations were restricted to CVR values measured from MCAv responses during which MAP remained unchanged.

### The effect of response dynamics on the measurement of CVR

To determine whether or not the speed of the response affected the CVR measured using the All and SS analyses, we examined the relative and absolute CVR values for differences when the response was classified as slow versus fast using a 2-way rmANOVA with factors analysis (All vs. SS) and speed of response (fast vs. slow).

**Figure 3:**
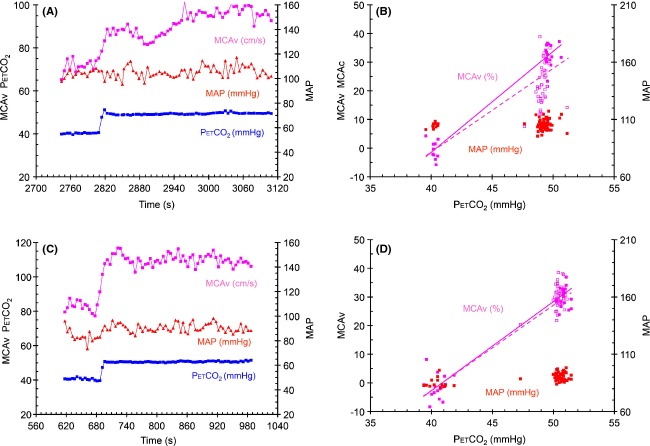
Step test results from subject 3 (A and B) with a slow oscillatory response, and subject 5 (C and D) with a fast response, both seated, showing breath-by-breath measures of PetCO_2_, MCAv and MAP. The graphs on the left show the time course of the variables and the graphs on the right show the relative MCAv and MAP responses to PetCO_2_. The lines in these graphs show the analyses used to calculate CVR. The SS analysis is indicated by the solid line and filled symbols, and the All analysis is indicated by the dotted line and open symbols. The axes for each variable are as indicated and the units are noted with the variable color-coded labels.

The All and SS CVR values differed for slow responses (*P* ≤ 0.001 and 0.002 for relative and absolute CVR, respectively), but did not differ for the fast responses (*P* = 0.837 and 0.806 for relative and absolute CVR, respectively), as illustrated in Figure 3.

### The effect of position (sitting vs. supine) on the measurement of CVR

We, therefore, tested position as a significant factor for only the SS MCAv responses where MAP was constant using a 2-way rmANOVA with the factors measurement type (Rel vs. Abs) and position (sitting vs. supine). Table 5 shows that although the CVR was lower in the sitting position, it was not significantly so. However, absolute CVR values were less than that of relative CVR values whether seated or supine.

**Table 5:** Mean (SD) CVR calculated from the MCAv steady state (SS) relative (Rel, %/mmHg) and absolute (Abs, cm/s/mmHg) responses during constant MAP in different positions (sitting and supine). The corresponding *P* values are the result of 2-way rmANOVA testing with all pairwise multiple comparisons using the Holm–Sidak method. Significantly different measures are bold and italicized

Position	Rel	Abs	*P*
Supine	4.09 (1.28)	3.18 (1.40)	***<0.001***
Sitting	3.10 (0.35)	2.18 (0.69)	***0.003***
*P*	0.092	0.169	

### CVR determined from ramp increases in CO_2_

**Figure 4:**
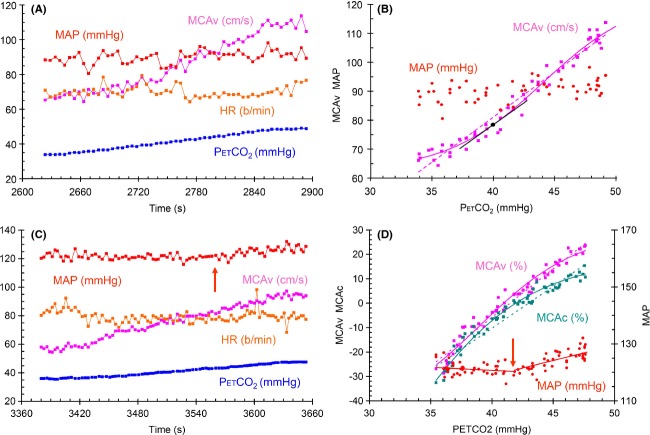
(A, B) Ramp test results for subject 6 seated showing breath-by-breath measures of PetCO_2_, MCAv, HR and MAP. Graph A on the left shows the time course of the variables and graph B on the right shows the absolute MCAv and MAP responses to PetCO_2_. The lines in graph B show the analyses used to calculate CVR. The Sig analysis is indicated by the solid purple line, with the baseline PetCO_2_-point marked as a circle and the tangent to the sigmoid curve at that point marked as a black line, and the Lin analysis is indicated by the dotted line. (C, D) Ramp test results from subject 4 seated. Graph A on the left shows the breath-by-breath measures of PetCO_2_, MCAv, MCAc, MAP and HR during the ramp change in PetCO_2_. Graph B on the right shows the relative MCAv, MCAc and the MAP responses to PetCO_2_, with the latter showing the sub and superthreshold linear fits to the data. The MAP threshold is indicated by arrows on each graph. In graph B, the solid lines show the fitted sigmoidal curve of the Sig analysis and the dotted lines, the linear regression of the Lin analysis. The axes for each variable are as indicated and the units are noted with the color-coded variable labels.

The MCAv responses were fitted with either a straight line or a sigmoid as illustrated in Figure 4A and B; with the slope of the straight line taken as the Lin CVR and the slope of a tangent to the sigmoid curve at the baseline PetCO_2_ as the Sig CVR. We examined the changes in MAP during the ramp increase in CO_2_ by fitting two linear segments above and below a threshold where the slope changed abruptly. Figure 4C and D illustrates the effect and shows that the MCAv and MCAc responses diverged markedly above the threshold PetCO_2_ where MAP began to increase. Ramp tests where the sub-threshold slope was > 0.6 mmHg/mmHg and the threshold < 46 mmHg were eliminated from further analysis as confounded by the concurrent increase in MAP. Table 6 records the CVR values calculated for ramp MCAv response to CO_2_ where MAP was constant.

**Table 6:** Ramp response measures of relative (Rel, %/mmHg) and absolute (Abs, cm/s/mmHg) CVR values using the linear and sigmoid analyses of the MCAv responses

		Linear		Sigmoid	
			
Subject	Position	Rel	Abs	Rel	Abs
S2	Supine	3.28	1.75	4.10	2.08
S2	Sitting	3.40	1.94	3.18	1.81
S3	Supine	6.00	4.27	4.04	4.54
S3	Sitting	3.68	2.67	4.04	2.81
S4	Sitting	4.30	3.31	4.63	3.43
S5	Supine	3.36	2.79	2.76	2.41
S6	Supine	4.56	3.93	4.88	4.04
S6	Sitting	3.94	3.14	3.57	2.80
S8	Supine	4.48	3.44	3.55	4.28
S8	Sitting	3.48	2.89	3.79	3.35
S9	Supine	3.79	2.20	3.49	2.31
S9	Sitting	3.42	1.31	2.26	1.77

### The effect of position and fitting function on the measurement of CVR

These CVR measures were examined to determine if the linear and sigmoid fitting of the responses produced different CVR values, and to see if position was a significant factor using a 2-way rmANOVA with factors analysis (linear vs. sigmoid) and position (sitting vs. supine). Table 7 shows that the linear and sigmoidal analyses produced CVR values that were not different (*P* = 0.153 and 0.198 for relative and absolute values, respectively). Furthermore, position was not a significant factor despite the finding of a decreased CVR in the sitting position (*P* = 0.697 and 0.490 for relative and absolute values, respectively). A 1-way rmANOVA comparison of the sitting vs. supine sigmoidal characteristics also found no difference between sitting and supine tests.

**Table 7:** Mean (SD) CVR calculated from the MCAv relative (Rel, %/mmHg) and absolute (Abs, cm/s/mmHg) responses during constant MAP in different positions (sitting and supine) using linear and sigmoidal analyses

	Rel		Abs	
		
Position	Linear	Sigmoidal	Linear	Sigmoidal
Supine	4.21 (0.46)	3.56 (1.31)	3.12 (1.20)	3.36 (1.38)
Sitting	3.53 (0.49)	3.62 (0.15)	2.54 (0.49)	2.82 (0.52)

### Comparing CVR from step and ramp stimuli

We compared the CVR values obtained from the linear analysis of the ramp MCAv responses with their corresponding CVR values obtained from the SS analysis of the step MCAv responses (tests where MAP was not a factor) using a 2-way rmANOVA with factors test stimulus type (step vs. ramp) and relative (Rel, %/mmHg) versus absolute (Abs, cm/s/mmHg) units. Table 8 shows that the step CVR values were smaller than the ramp CVR values regardless of whether in relative or absolute units, and that the absolute CVR values were smaller than the relative CVR values regardless of the stimulus pattern.

**Table 8:** Mean (SD) CVR calculated from the MCAv steady state (SS) relative (Rel, %/mmHg) and absolute (Abs, cm/sec per mmHg) responses to step and ramp stimulus patterns during constant MAP. The corresponding *P* values are the result of 2-way rmANOVA testing with all pairwise multiple comparisons using the Holm–Sidak method. Significantly different measures are bolded and italicised

Stimulus	Rel	Abs	*P*
Step	3.53 (1.01)	2.48 (0.94)	***<0.001***
Ramp	3.94 (0.81)	2.76 (0.91)	***<0.001***
*P*	***0.005***	***0.040***	

### Baseline PetCO_2_ and the PetCO_2_ of maximum CVR

Finally, the sigmoidal fitting of the responses also provided a measure of the PetCO_2_ at which the sigmoidal response slope was the highest (the midpoint parameter). A 1-way rmANOVA analysis showed that the midpoints of the sigmoid functions fitted to the relative MCAv responses (mean [SD] = 42.5 [2.6] mmHg) were not different from those of the absolute responses (mean [SD] = 41.7 [3] mmHg), but both were higher than the baseline PetCO_2_ of these selected tests (mean [SD] = 39.9 [0.8] mmHg). The baseline PetCO_2_ chosen for these experiments was, therefore, slightly lower than the PetCO_2_ where CVR is a maximum and the cerebrovasculature midway between its limits of vasoconstriction and vasodilation.

### CVR determined from transfer function analysis

**Figure 5:**
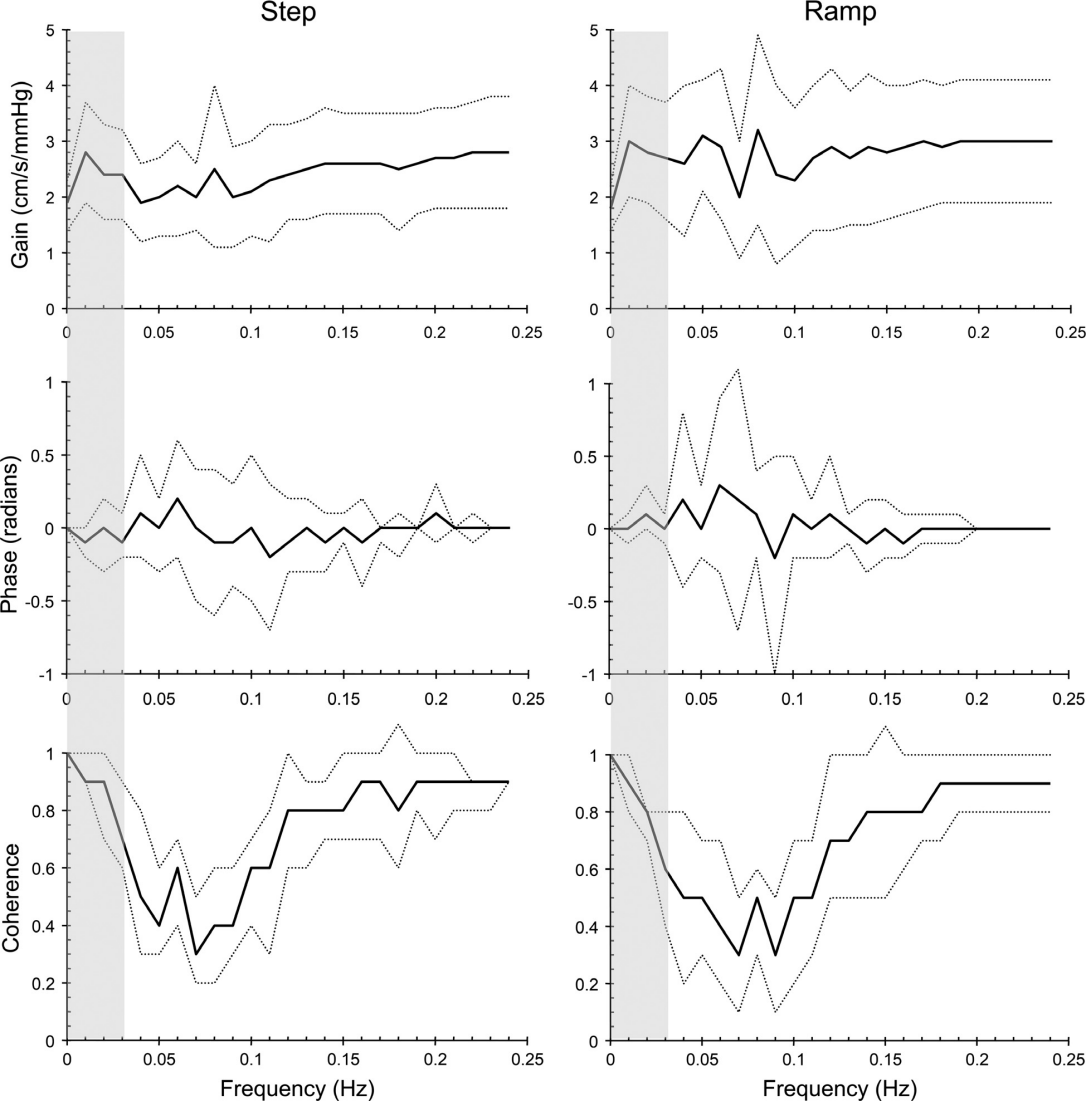
Ensemble averages (thick lines) ± SD (thin lines) for Gain, Phase and Coherence from ramp and step responses with MAP constant. The shaded area indicates the frequency band used to calculate the mean Gain = TFA CVR.

**Figure 6:**
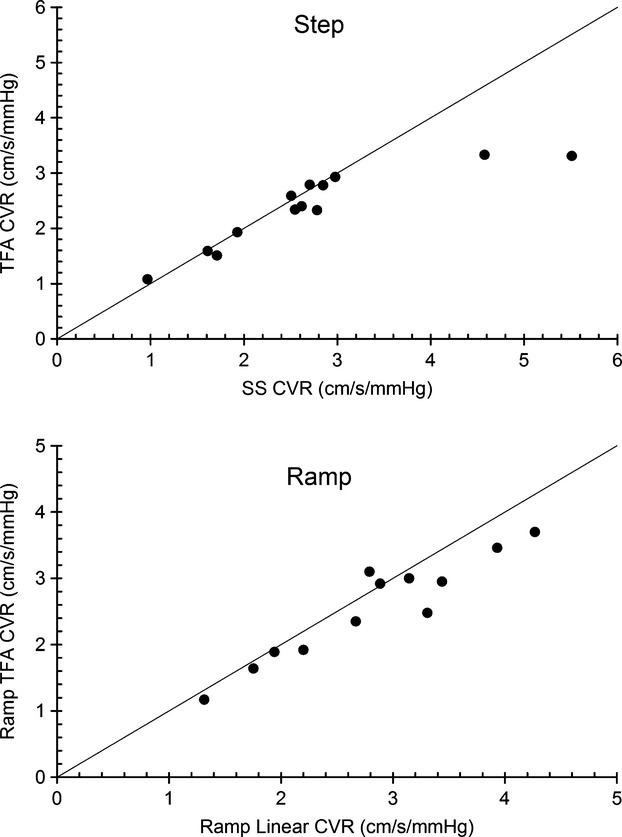
The relationship between CVR calculated from the SS step responses and the Lin ramp responses with constant MAP and CVR calculated from transfer function analysis (TFA). The line of equality is shown.

We examined the ramp and step MCAv (cm/s/mmHg) responses where MAP was not a confounding factor with transfer function analysis. Figure 5 shows the ensemble averages for Gain, Phase, and Coherence. Figure 6 shows the relation between CVR calculated from the linear fit to the ramp responses and from the steady-state step responses versus TFA CVR. The Pearson product moment correlation coefficients were 0.942 and 0.892 for the ramp and step tests, respectively.

We noted that the phase reflected the speed of the response; the mean (SD) phase of 6 step responses classified as slow or oscillatory was −0.27 (0.15) radians compared to 0.01 (0.05) radians for the fast responses.

## Discussion

### General

These experiments were undertaken with a view to examining some factors that could affect the measurement of CVR. We use the term CVR in the sense of a vascular response to CO_2_, i.e. vascular diameter changes. It is this aspect that assumes clinical importance in defining the ability of a vascular bed to adjust its flow resistance to cope with challenges to its blood flow supply. Such cerebrovascular reactivity measurements to assess cerebrovascular health have important clinical applications. Impaired vascular reactivity has been linked to an increased risk of stroke (Kleiser and Widder 1992; Yonas et al. 1993; Webster et al. 1995; Molina et al. 1999; Markus and Cullinane 2001; Ogasawara et al. 2002; Sasoh et al. 2003), and indeed, areas of reduced cerebral reactivity, such that steal occurs during hypercapnia, are those where leukoaraiosis occurs. Cortical thinning results in areas of poor vascular reactivity (Fierstra et al. 2010), and in their recent review Marshall and Lazar (2011) suggest that cognitive impairment may be independently mediated by hemodynamic dysfunction. There is evidence for an association between cognitive dysfunction and hemodynamics impairment due to carotid stenosis (Balucani et al. 2012; Balestrini et al. 2013), and alterations of cerebral vessel functional and anatomic status have been shown with dementia (Marshall and Lazar 2011; Silvestrini et al. 2011), and to increase the risk of conversion from mild cognitive impairment to dementia (Viticchi et al. 2012).

We used step and ramp stimulus patterns to obtain the CVR values that are in common use so as to highlight the possible pitfalls involved in measuring CVR, and introduced an alternative method of calculating CVR using transfer function analysis. We suggest that the most serious pitfall is ignoring blood pressure increases with CO_2_. Indeed, we recommend that tests with such increases in blood pressure be discarded as measures of the vascular diameter changes with CO_2_ for reasons which we discuss below. The second aspect of CVR measurement often neglected is the effect of the stimulus and response patterns on CVR; these must be taken into consideration when choosing the analysis of the blood flow responses. Finally, although we suspected that body position might affect the CVR, we nevertheless found that in these experiments it did not. These aspects of CVR measurement are discussed in detail in the following sections.

### Blood pressure

In tests where MAP increases with CO_2_, the question arises as to whether the blood flow measurement can be used to correctly assess the vasoactive response to CO_2_. Some authors suggest that measuring the response in terms of MCAc eliminates the confounding effect of changes in perfusion pressure e.g. (Claassen et al. 2007; Willie et al. 2012). Alternatively, CVR can be taken to include any changes due to MAP, and thereby represent the CBF response that would be observed for environmental hypercapnia such as occurring in obstructive sleep apnea or as adaptation to altitude (Fan et al. 2014). However, we argue that if CVR is used to assess vascular reactivity in terms of vessel flow resistance, for example, to detect pathophysiology (Mandell et al. 2008b; Sobczyk et al. 2014), then increases in perfusion pressure during the test are a confounding factor.

It is likely that cerebral perfusion pressure increases when MAP increases. In that case, CBF is determined by a number of concurrently operating factors as follows: (1) CO_2_ vasodilation; (2) pressure autoregulation vasconstriction; (3) physical pressure vasodilation; (4) physical passive pressure flow increase. Pressure autoregulation vasoconstriction and CO_2_ vasodilation interact since both adjust vessel diameter, so that if autoregulation is not exhausted, then any increase in perfusion pressure invokes autoregulation adjustment of cerebrovascular conductance to regulate cerebral blood flow (Baumbach and Heistad 1983). Even if autoregulation is exhausted, CBF increases not only due to the vasodilatory effect of CO_2_ but also due to the direct effect of perfusion pressure on flow (Battisti-Charbonney et al. 2011).

In none of these circumstances is it possible to separate the effects of perfusion pressure and CO_2_-induced vasodilation on cerebral blood flow. While MCAc in these experiments does provide an estimate of the conductance response to CO_2_ due to the various factors involved, it does not estimate the vasodilatory effect of CO_2_, and so using the MCAc response to measure CVR is misleading. We concluded that increases in MAP with CO_2_ remain a confounding factor when measuring CVR, and consequently responses where MAP increased were excluded from further analysis in this study.

We note that blood pressure increases with CO_2_ that confound cerebral blood flow responses to CO_2_ are routinely disregarded in experiments such as breath-holding (e.g. Bright and Murphy 2013) despite possible confounding increases in MAP (Przybylowski et al. 2003), which may be substantial (Cummings et al. 2007). Similarly, MAP increases with increasing CO_2_ produced by rebreathing (Vovk et al. 2002; Claassen et al. 2007; Battisti-Charbonney et al. 2011) or CO_2_ inhalation (Hetzel et al. 1999; Valdueza et al. 1999) are often ignored (e.g. Thomas et al. 2013) when measuring CVR. We suggest that blood pressure be monitored during CVR testing and those tests where MAP increases be rejected if using CBF to assess the vasoactive response to CO_2_. These tests measure the CBF response to CO_2_ not the reactivity of the cerebral vasculature.

We also noted the changes in HR for those subjects that increased MAP in response to CO_2_. For the step stimulus, the HR response was mixed, with one subject increasing HR as MAP increased (subject 4 sitting and supine) and other subjects decreasing HR (subjects 1 sitting, and 7 sitting and supine). The subjects with MAP increases with CO_2_ during ramp stimuli and showed no discernible concurrent changes in HR. Although these changes in heart rate may indicate changes in sympathetic and parasympathetic tone (Peebles et al. 2012), there is insufficient information in these experiments to make a definitive interpretation.

### Step versus ramp

We measured CVR using both a step increase in PetCO_2_ and a ramp increase; the ramp stimulus providing a slow change in CO_2_ in contrast to the rapid increase of the step stimulus. Several characteristics of these stimuli affect the CBF response. First, since the CBF response to CO_2_ is sigmoidal (Battisti-Charbonney et al. 2011), both the range and starting PetCO_2_ determine the CBF response (Sobczyk et al. 2014). A stimulus that impinges on the upper or lower limits will necessarily produce an attenuated response and lower CVR. Indeed, we attribute the lower CVR measured for the step stimulus compared to that measured for the ramp stimulus due to that effect. The ramp stimulus range included both hypocapnic and hypercapnic PetCO_2_, so that it bracketed resting PetCO_2_ where CVR is maximum, whereas the step stimulus was limited to the hypercapnic range.

The ramp stimulus range in these experiments, where PetCO_2_ varied from approximately 35 to 50 mmHg, was less than that used in our previous rebreathing experiments, where PetCO_2_ ranged from 25 to 55 mmHg (Battisti-Charbonney et al. 2011). With such a limited stimulus range, the response appeared to be within the linear portion of the response in most tests. However, in some ramp tests, the response showed a degree of limitation at the minimum or maximum stimulus levels such that the sigmoidal fit appeared more appropriate than a linear fit as illustrated in Figure 4B. Nevertheless, we found that Lin and Sig CVR measures were similar in these experiments, and concluded that with a limited stimulus range, both linear and sigmoidal fitting to ramp responses can be used to determine CVR. We note however, that the sigmoidal analysis provides additional information (the upper and lower limits of vasodilation and constriction, the linear range and the Pco_2_ at which CVR is maximum) and should be used when the stimulus range is large.

The rapid changes in PetCO_2_ produced by the step stimulus elicited responses that revealed their dynamic aspects; in these experiments, we were able to discern slow and fast responses as well as two oscillatory responses as previously observed (Regan et al. 2013). As our analysis showed, the CVR values calculated using all of the hypercapnic response were less than the CVR values calculated using the later steady-state section of the hypercapnic response for slow responses. This low CVR value may be useful for discriminating a pathophysiology involving slow cerebrovascular dynamics, and is often used in the calculation of CVR maps using BOLD MRI (e.g. Mandell et al. 2008a).

The decision to use a step or ramp stimulus therefore involves consideration of the advantages and disadvantages of each, and the previous results and discussion enables such an enumeration. If the dynamics of the response are an important aspect of the CVR measurement, then we suggest choosing the step stimulus, and the appropriate portion of the response used for analysis. For example, CVR determined for slow responses analyzed using all of the response was less than CVR calculated from the steady-state portion of the response, and also less than CVR calculated from a ramp response. This low CVR value may be useful for discriminating a pathophysiology involving slowed cerebrovascular dynamics.

The main disadvantage of the step stimulus is that only two points on the entire response are measured so the portion of the sigmoidal response that is measured is unknown. For example, CVR measured with a stimulus range that includes the upper limits of the sigmoidal response will be less than CVR measured with a stimulus range that includes the midrange of the sigmoidal response. This disadvantage of the step stimulus is overcome by using a ramp stimulus so that the sigmoidal nature of the response can be observed and the linear portion identified. However, the ramp response does not examine the response dynamics. There is a final aspect contrasting step and ramp stimuli, that of subject tolerance. In our experience, the ramp response with its slow increase in hypercapnia and short exposure to the peak stimulus is better tolerated than the rapid step increase in hypercapnia whose peak is maintained longer.

### Relative versus absolute measures of CVR

In these experiments, the baseline MCAv did not differ between the various conditions (sitting vs. supine) and the ramp and step stimuli. Therefore, the statistical test results between relative and absolute CVR measures did not differ. However, in cases where the baseline CBF values differ between conditions, the choice between relative and absolute CVR measures may affect the statistical test outcome, since changes in baseline CBF inversely affect the relative CVR.

### Transfer function analysis

In these experiments, we introduced the use of transfer function analysis to calculate CVR, limiting the analysis to the ramp and step responses where MAP was constant. The TFA CVR values were similar to those calculated from the linear fits to the ramp responses and the steady-state step responses, and well correlated. Moreover, the phase values correlated with the speed of response determined from exponential fitting. We therefore concluded that TFA analysis deserves further investigation, and in particular we suggest that TFA analysis may be useful in BOLD MRI mapping of CVR, where the phase response could indicate areas of slowed responsiveness.

## Conclusions

These experiments, using MCAv as a surrogate for CBF and CO_2_ as a stimulus, led to several conclusions bearing on the measurement of CVR. First, if the measurements of CVR are to be used as an indication of cerebrovascular vasodilatory reactivity, then MAP must be monitored, and CVR should be calculated from the change in MCAv while MAP remains constant to avoid the confounding action of changes in MAP with CO_2_. Second, when CVR is measured from the responses to a step stimulus pattern from baseline to hypercapnia, it is important to observe the dynamics of the response and use an analysis appropriate to the study aim. Third, if the ramp stimulus range is limited, then CVR values calculated from linear regression are equivalent to those calculated from fitting a sigmoidal function to the response, but the sigmoidal function parameters offer additional information. Fourth, transfer function analysis to calculate CVR appears to be a technique worthy of further investigation. Fifth, the subject position, sitting or supine did not alter the CVR. Finally, CVR calculated from relative responses differ from those calculated from absolute responses, but did not alter the statistical test results in these experiments.

## References

[b1] Ainslie PN, Burgess KR (2008). Cardiorespiratory and cerebrovascular responses to hyperoxic and hypoxic rebreathing: effects of acclimatization to high altitude. Respi. Physiol. Neurobiol.

[b2] Attwell D, Buchan AM, Charpak S, Lauritzen M, Macvicar BA, Newman EA (2011). Glial and neuronal control of brain blood flow. Nature.

[b3] Balestrini S, Perozzi C, Altamura C, Vernieri F, Luzzi S, Bartolini M (2013). Severe carotid stenosis and impaired cerebral hemodynamics can influence cognitive deterioration. Neurology.

[b4] Balucani C, Viticchi G, Falsetti L, Silvestrini M (2012). Cerebral hemodynamics and cognitive performance in bilateral asymptomatic carotid stenosis. Neurology.

[b5] Battisti-Charbonney A, Fisher J, Duffin J (2011). The cerebrovascular response to carbon dioxide in humans. J. Physiol.

[b6] Baumbach GL, Heistad DD (1983). Effects of sympathetic stimulation and changes in arterial pressure on segmental resistance of cerebral vessels in rabbits and cats. Circ. Res.

[b7] Bright MG, Murphy K (2013). Reliable quantification of BOLD fMRI cerebrovascular reactivity despite poor breath-hold performance. Neuroimage.

[b8] Claassen JA, Zhang R, Fu Q, Witkowski S, Levine BD (2007). Transcranial Doppler estimation of cerebral blood flow and cerebrovascular conductance during modified rebreathing. J. Appl. Physiol.

[b9] Conklin J, Fierstra J, Crawley AP, Han JS, Poublanc J, Mandell DM (2010). Impaired cerebrovascular reactivity with steal phenomenon is associated with increased diffusion in white matter of patients with Moyamoya disease. Stroke.

[b10] Cummings KJ, Swart M, Ainslie PN (2007). Morning attenuation in cerebrovascular CO_2_ reactivity in healthy humans is associated with a lowered cerebral oxygenation and an augmented ventilatory response to CO_2_. J. Appl. Physiol.

[b11] Dineen NE, Brodie FG, Robinson TG, Panerai RB (2010). Continuous estimates of dynamic cerebral autoregulation during transient hypocapnia and hypercapnia. J. Appl. Physiol.

[b12] Fan J-L, Subudhi AW, Evero O, Bourdillon N, Kayser B, Lovering AT (2014). AltitudeOmics: enhanced cerebrovascular reactivity and ventilatory response to CO_2_ with high-altitude acclimatization and reexposure. J. Appl. Physiol.

[b13] Fierstra J, Poublanc J, Han JS, Silver F, Tymianski M, Crawley AP (2010). Steal physiology is spatially associated with cortical thinning. J. Neurol. Neurosurg. Psychiatry.

[b14] Fierstra J, Sobczyk O, Battisti-Charbonney A, Mandell DM, Poublanc J, Crawley AP (2013). Measuring cerebrovascular reactivity: what stimulus to use?. J. Physiol.

[b15] Han JS, Abou-Hamden A, Mandell DM, Poublanc J, Crawley AP, Fisher JA (2011). Impact of extracranial-intracranial bypass on cerebrovascular reactivity and clinical outcome in patients with symptomatic moyamoya vasculopathy. Stroke.

[b16] Harper AM, Glass HI (1965). Effect of alterations in the arterial carbon dioxide tension on the blood flow through the cerebral cortex at normal and low arterial blood pressures. J. Neurol. Neurosurg. Psychiatry.

[b17] Hetzel A, Braune S, Guschlbauer B, Dohms K (1999). CO_2_ reactivity testing without blood pressure monitoring?. Stroke.

[b18] Iadecola C, Nedergaard M (2007). Glial regulation of the cerebral microvasculature. Nat. Neurosci.

[b19] Ito S, Mardimae A, Han J, Duffin J, Wells G, Fedorko L (2008). Non-invasive prospective targeting of arterial PCO_2_ in subjects at rest. J. Physiol.

[b20] Kleiser B, Widder B (1992). Course of carotid artery occlusions with impaired cerebrovascular reactivity. Stroke.

[b21] Kontos HA, Wei EP, Raper AJ, Patterson JL (1977). Local mechanism of CO_2_ action of cat pial arterioles. Stroke.

[b22] Lassen NA (1968). Brain extracellular pH: the main factor controlling cerebral blood flow. Scand. J. Clin. Lab. Invest.

[b23] Lucas SJ, Tzeng YC, Galvin SD, Thomas KN, Ogoh S, Ainslie PN (2010). Influence of changes in blood pressure on cerebral perfusion and oxygenation. Hypertension.

[b24] Mandell DM, Han JS, Poublanc J, Crawley AP, Kassner A, Fisher JA (2008a). Selective reduction of blood flow to white matter during hypercapnia corresponds with leukoaraiosis. Stroke.

[b25] Mandell DM, Han JS, Poublanc J, Crawley AP, Stainsby JA, Fisher JA (2008b). Mapping cerebrovascular reactivity using blood oxygen level-dependent MRI in patients with arterial steno-occlusive disease: comparison with arterial spin labeling MRI. Stroke.

[b26] Markus H, Cullinane M (2001). Severely impaired cerebrovascular reactivity predicts stroke and TIA risk in patients with carotid artery stenosis and occlusion. Brain.

[b27] Marshall RS, Lazar RM (2011). Pumps, aqueducts, and drought management: vascular physiology in vascular cognitive impairment. Stroke.

[b28] McDonnell MN, Berry NM, Cutting MA, Keage HA, Buckley JD, Howe PR (2013). Transcranial Doppler ultrasound to assess cerebrovascular reactivity: reliability, reproducibility and effect of posture. PeerJ.

[b29] Molina C, Sabin JA, Montaner J, Rovira A, Abilleira S, Codina A (1999). Impaired cerebrovascular reactivity as a risk marker for first-ever lacunar infarction: a case-control study. Stroke.

[b30] Ogasawara K, Ogawa A, Yoshimoto T (2002). Cerebrovascular reactivity to acetazolamide and outcome in patients with symptomatic internal carotid or middle cerebral artery occlusion: a xenon-133 single-photon emission computed tomography study. Stroke.

[b31] Panerai RB, Evans DH, Naylor AR (1999). Influence of arterial blood pressure on cerebrovascular reactivity. Stroke.

[b32] Paulson OB, Hasselbalch SG, Rostrup E, Knudsen GM, Pelligrino D (2011). Cerebral blood flow response to functional activation. J. Cereb. Blood Flow Metab.

[b33] Peebles KC, Ball OG, MacRae BA, Horsman HM, Tzeng YC (2012). Sympathetic regulation of the human cerebrovascular response to carbon dioxide. J. Appl. Physiol.

[b34] Poulin MJ, Liang PJ, Robbins PA (1996). Dynamics of the cerebral blood flow response to step changes in end- tidal PCO_2_ and PO_2_ in humans. J. Appl. Physiol.

[b35] Przybylowski T, Bangash M-F, Reichmuth K, Morgan BJ, Skatrud JB, Dempsey JA (2003). Mechanisms of the cerebrovascular response to apnoea in humans. J. Physiol.

[b36] Regan RE, Duffin J, Fisher JA (2013). Instability of the middle cerebral artery blood flow in response to CO_2_. PLoS One.

[b37] Sasoh M, Ogasawara K, Kuroda K, Okuguchi T, Terasaki K, Yamadate K (2003). Effects of EC-IC bypass surgery on cognitive impairment in patients with hemodynamic cerebral ischemia. Surg. Neurol.

[b38] Silvestrini M, Viticchi G, Falsetti L, Balucani C, Vernieri F, Cerqua R (2011). The role of carotid atherosclerosis in Alzheimer's disease progression. J. Alzheimers Dis.

[b39] Slessarev M, Han J, Mardimae A, Prisman E, Preiss D, Volgyesi G (2007). Prospective targeting and control of end-tidal CO_2_ and O_2_ concentrations. J. Physiol.

[b40] Sobczyk O, Battisti-Charbonney A, Fierstra J, Mandell DM, Poublanc J, Crawley AP (2014). A conceptual model for CO_2_-induced redistribution of cerebral blood flow with experimental confirmation using BOLD MRI. NeuroImage.

[b41] Thomas BP, Liu P, Aslan S, King KS, van Osch MJ, Lu H (2013). Physiologic underpinnings of negative BOLD cerebrovascular reactivity in brain ventricles. Neuroimage.

[b42] Tian R, Vogel P, Lassen NA, Mulvany MJ, Andreasen F, Aalkjaer C (1995). Role of extracellular and intracellular acidosis for hypercapnia-induced inhibition of tension of isolated rat cerebral arteries. Circ. Res.

[b43] T-M W, Lu LC, Ye XL, Li S, Wang LX (2008). Impact of postures on blood pressure in healthy subjects. Acta Clin. Belg.

[b44] Totaro R, Marini C, Baldassarre M, Carolei A (1999). Cerebrovascular reactivity evaluated by transcranial Doppler: reproducibility of different methods. Cerebrovasc. Dis.

[b45] Tzeng YC, Ainslie PN, Cooke WH, Peebles KC, Willie CK, MacRae BA (2012). Assessment of cerebral autoregulation: the quandary of quantification. Am. J. Physiol. Heart Circ. Physiol.

[b46] Valdueza JM, Draganski B, Hoffmann O, Dirnagl U, Einhaupl KM (1999). Analysis of CO_2_ vasomotor reactivity and vessel diameter changes by simultaneous venous and arterial Doppler recordings. Stroke.

[b47] Viticchi G, Falsetti L, Vernieri F, Altamura C, Bartolini M, Luzzi S (2012). Vascular predictors of cognitive decline in patients with mild cognitive impairment. Neurobiol. Aging.

[b48] Vovk A, Cunningham DA, Kowalchuk JM, Paterson DH, Duffin J (2002). Cerebral blood flow responses to changes in oxygen and carbon dioxide in humans. Can. J. Physiol. Pharmacol.

[b49] Webster MW, Makaroun MS, Steed DL, Smith HA, Johnson DW, Yonas H (1995). Compromised cerebral blood flow reactivity is a predictor of stroke in patients with symptomatic carotid artery occlusive disease. J. Vasc. Surg.

[b50] Willie CK, Colino FL, Bailey DM, Tzeng YC, Binsted G, Jones LW (2011). Utility of transcranial Doppler ultrasound for the integrative assessment of cerebrovascular function. J. Neurosci. Methods.

[b51] Willie CK, Macleod DB, Shaw AD, Smith KJ, Tzeng YC, Eves ND (2012). Regional brain blood flow in man during acute changes in arterial blood gases. J. Physiol.

[b52] Willie CK, Tzeng YC, Fisher JA, Ainslie PN (2014). Integrative regulation of human brain blood flow. J. Physiol.

[b53] Yonas H, Smith HA, Durham SR, Pentheny SL, Johnson DW (1993). Increased stroke risk predicted by compromised cerebral blood flow reactivity. J. Neurosurg.

